# Preliminary outcomes of the surgical navigation system combined with intraoperative three-dimensional C-arm computed tomography for zygomatico-orbital fracture reconstruction

**DOI:** 10.1038/s41598-022-11659-x

**Published:** 2022-05-12

**Authors:** Yu-Ying Chu, Jia-Ruei Yang, Bo-Ru Lai, Han-Tsung Liao

**Affiliations:** 1 Division of Trauma Plastic Surgery, Department of Plastic and Reconstructive Surgery, Craniofacial Research Center, Chang Gung Memorial Hospital, College of Medicine, Chang Gung University, 5 Fuxing Street, Taoyuan, 333 Taiwan; 2 Craniofacial Research Center, Chang Gung Memorial Hospital, Taoyuan, 333 Taiwan; 3 College of Medicine, Chang Gung University, Taoyuan, 333 Taiwan; 4 Department of Plastic Surgery, Xiamen Chang Gung Hospital, Xiamen, 361000 China

**Keywords:** Anatomy, Medical research

## Abstract

This study analyzed the outcomes of zygomatico-orbital fracture reconstruction using the real-time navigation system with intraoperative three-dimensional (3D) C-arm computed tomography (CT). Fifteen patients with zygomatico-orbital or isolated orbital/zygoma fractures were enrolled in this prospective cohort. For zygoma reduction, the displacement at five key sutures and the differences between preoperative and intraoperative CT images were compared. For orbital reconstruction, the bilateral orbital volume differences in the anterior, middle, and posterior angles over the medial transitional buttress were measured. Two patients required implant adjustment once after the intraoperative 3D C-arm assessment. On comparing the preoperative and postoperative findings for the zygoma, the average sum of displacement was 19.48 (range, 5.1–34.65) vs. 1.96 (0–3.95) mm (*P* < 0.001) and the deviation index was 13.56 (10–24.35) vs. 2.44 (0.6–4.85) (*P* < 0.001). For the orbit, the mean preoperative to postoperative bilateral orbital volume difference was 3.93 (0.35–10.95) vs. 1.05 (0.12–3.61) mm^3^ (*P* < 0.001). The mean difference in the bilateral angles at the transition buttress was significantly decreased postoperatively at the middle and posterior one-third. There was no significant difference in orbital volume, angle of the transition zone, and the sum of five zygoma distances between post operative results and preoperative virtual planning. The surgical navigation system with the intraoperative 3D C-arm can effectively improve the accuracy of zygomatico-orbital fracture reconstruction and decrease implant adjustment times.

## Introduction

Zygomatico-orbital (ZMO) fractures refer to injuries of the zygoma and the surrounding bone, including the orbit^1^. They are the most common type of orbital fractures^2^. Without adequate reduction and reconstruction of ZMO fractures, complications such as malar flattening, facial asymmetry, sensory disturbance, enophthalmos, hypoglobus, and diplopia may occur^3,4^. Therefore, a computer-assisted surgical navigation system and intraoperative computer tomography (CT) have been frequently proposed in the literature for the management of facial fracture surgery^5–19^. The intraoperative navigation system provides accurate reduction of the fracture bone that matches the virtually simulated position that was planned preoperatively^6^. It helps in confirming the position of the orbital implant and reduction of the displaced zygomatic fracture, optimizing the functional and aesthetic outcomes of orbit fractures^10,20^, and achieving postoperative symmetrical facial profiles for patients with zygomaticomaxillary complex (ZMC) fractures^21^. Although it involves real-time guidance, the accuracy is based on point-to-point confirmation instead of a match to the three-dimensional (3D) anatomical architecture of reduced bone. On the other hand, intraoperative 3D C-arm CT has also been well applied in craniofacial reconstruction. It can evaluate the adequacy of ZMC fracture reduction^22^ and ensure the implant position in ZMO fractures or isolated orbital fractures via a match to 3D architecture^23^. However, the use of the 3D C-arm is difficult with real-time guidance, and the intraoperative revision rate can be higher than 63%^17^. Although the radiation dose of the intraoperative 3D C-arm CT is lower than that of conventional CT, frequent adjustments put the patient at risk of higher radiation exposure.

To combine the advantages and eliminate the disadvantages of these two techniques, our study aimed to determine the surgical outcomes of patients with ZMO fractures or isolated zygoma/orbital fractures who underwent reconstruction under the guidance of a surgical navigation system combined with the intraoperative 3D C-arm and demonstrate the practical potential of this technology to achieve the precise and accurate operative results.

## Results

### Demographics and symptoms

A total of 15 patients (4 males, 11 females) were enrolled in this study. The mean age was 39.2 ± 16.0 years. Seven patients had ZMO fractures (2 Type A, 4 Type B, 1 Type C)^24^, seven had pure orbital fracture, and one had an isolated zygoma fracture (Type B). The fractures occurred on the right side in eight patients and on the left in seven. Thirteen fractures resulted from traffic accidents, one from a fall, and one from a human altercation. Seven patients received early/primary reconstruction, three underwent late reconstruction, and five had a secondary revision. After zygoma reduction, the MatrixMIDFACE™ Plating System was used for fixation, and a preformed mesh (DePuy Synthes Matrix MIDFACE Preformed Orbital Plates), titanium mesh, and Medpor were used for reconstruction and to reinforce the support of the fractured orbital floor and the medial wall. Intraoperatively, two patients required one adjustment of the implant and fixation plate position after 3D C-arm assessment. There was no complication related to tool adapter fixation intraoperatively. On clinical presentation, five patients had preoperative diplopia, five had enophthalmos, two had hypoglobus, four had EOM limitation, and eight had facial asymmetry. One patient reported traumatic optic neuropathy that resulted in visual loss, causing difficulty in diplopia assessment. There were no postoperative cases of facial asymmetry. Diplopia persisted postoperatively in three of the five patients (one had enophthalmos, one had hypoglobus, and one had EOM limitation), but the situation improved from its preoperative status. The diplopia appeared postoperatively only when looking in a single direction instead of in multiple directions as observed preoperatively (Table 1).

**Table 1:** Patient demographics.

Characteristics	No. of patients n (%)
Age (y/o) [mean, (SD)]	39.2 (16.0)
**Sex**	
Male	4 (26.7)
Female	11 (73.3)
**Trauma mechanism**	
Traffic accident	13 (86.7)
Fall	1 (6.67)
Human altercation	1 (6.67)
**Fracture site**	
Right	8 (53.3)
Left	7 (46.7)
**Fracture pattern**	
Pure orbital	7 (46.7)
Pure zygoma	1 (6.7)
Zygomatico-orbital	7 (46.7)
**Surgical timing**	
Early	7 (46.7)
Late	3 (20)
Secondary	5 (33.3)
**Intraoperative adjustment**	
Yes	2 (13.3)
No	13 (86.6)
**Preoperative symptoms**	
Facial asymmetry	8 (53.3)
Diplopia	5 (33.3)
EOM limitation	4 (26.7)
Enophthalmos	5 (33.3)
Hypoglobus	2 (13.3)
**Postoperative symptoms**	
Facial asymmetry	0 (0)
Diplopia	3 (20)
EOM limitation	1 (6.7)
Enophthalmos	1 (6.7)
Hypoglobus	1 (6.7)

*EOM* extraocular muscle movement.

### Zygoma measurements

In 2D fashion, the mean displacement values at each zygomatic suture line preoperatively and postoperatively are shown in Table 2. The average sum of preoperative and postoperative displacement was 19.48 (range, 5.1–34.65) vs. 1.96 (0–3.95) mm (*P* < 0.001). In 3D fashion, the mean value of preoperative and postoperative differences between the surgical plan and CT images was also calculated. The deviation index (DI) preoperatively and postoperatively was 13.56 (10–24.35) vs. 2.44 (0.6–4.85) mm (*P* < 0.001) (Table 2). For the comparison between postoperative results and presurgical virtual plan, the mean value of sum of Z1O to Z5O and P1O to P5O were 339.69 (363–321.8) vs. 341.34 (359.9–316) (*P* = 0.63) mm. The result showed no statistically significant difference (Table 3).

**Table 2:** Preoperative and postoperative measurements for the zygoma.

	Preoperative finding (mm)						Postoperative finding (mm)						*P* value
	ZM	ZF	IO	ZS	ZT	Sum	ZM	ZF	IO	ZS	ZT	Sum	
Mean	7.43	1.91	4.78	4.49	0.88	19.48	0.09	0.00	0.07	1.79	0.00	1.96	< 0.001
Max	14.6	3.95	15.1	9.6	3.3	34.65	0.75	0	0.55	3.95	0	3.95	
Min	0	0	0	0	0	5.1	0	0	0	0	0	0	

	Preoperative finding (mm)						Postoperative finding (mm)						*P* value
	Z1P1	Z2P2	Z3P3	Z4P4	Z5P5	DI	Z1P1	Z2P2	Z3P3	Z4P4	Z5P5	DI	
Mean	5.14	3.57	2.15	1.16	1.54	13.56	0.69	0.56	0.13	0.34	0.71	2.44	< 0.001
Max	10.5	7.2	4.15	2.9	3.8	24.35	2	1.6	0.45	1.3	1.5	4.85	
Min	2	0.9	0.65	0	0.4	10	0	0	0	0	0.2	0.6	

*ZF* Zygomaticofrontal, *IO* Inferior orbital rim, *ZS* Zygomaticosphenoidal, *ZM* Zygomaticomaxillary, *ZT* Zygomaticotemporal, *SD* standard deviation, *Z* point on zygoma surface of CT image, *P* point on surface of zygoma object of presurgical planning, *DI* deviation index, *Max* maximum, *Min* minimum.

**Table 3:** Comparison of postoperative result and presurgical virtual plan.

	Postoperative result	Presurgical virtual plan	*P *value
**Orbit (n = 14)**			
Volume (mm^3^)*^,†^	26.21 (20.87–30.74)	26.06 (21.53–30.86)	0.87
Angle of TZ (degree)*^,†^			
Anterior 1/3	133.6 (116.6–147.2)	133.3 (114.3–146.7)	0.53
Middle	132.3 (115.1–143.5)	131.6 (105.7–149.6)	0.87
Posterior 1/3	138.6 (119.8–160.9)	141.2 (120.3–155.2)	0.58
**Zygoma (n = 8)**			
SUM (mm)	339.69 (321.8–363)	341.34 (316–359.9)	0.63

*SUM* sum of Z1O to Z5O or P1O to P5O; *Mean, ^†^Range.

### Orbit measurement

The mean values of the preoperative and postoperative bilateral orbital volume difference were 3.93 (0.35–10.95) vs. 1.05 (0.12–3.61) mm^3^ (*P* < 0.001). The mean values of the bilateral angle difference at the transition zone of the orbital buttress preoperatively and postoperatively were 15.94 (0.1–89) vs. 5.51 (0–30.1) (*P* = 0.134) degrees at the anterior one-third, 35.94 (0.5–144) vs. 5.74 (0.3–21.4) (*P* = 0.016) degrees at the middle, and 41.18 (5.1–127.3) vs. 5.15 (0–18) (*P* = 0.002) degrees at the posterior one-third, respectively (Table 4). For the comparison between postoperative results and presurgical virtual plan, the mean value of orbital volume revealed 26.21 (30.74–20.87) vs. 26.06 (30.86–21.53) (*P* = 0.87) mm^3^. The mean value of angle at the orbital transition buttress showed 133.6 (147.2–116.6) vs. 133.3 (146.7–114.3) (*P* = 0.53) degrees at the anterior one-third, 132.3 (143.5–115.1) vs. 131.6 (149.6–105.7) (*P* = 0.87) degrees at the middle and 138.6 (160.9–119.8) vs. 141.2 (155.2–120.3) (*P* = 0.58) degrees at the posterior one-third, respectively. All the results showed no statistically significant difference (Table 3).

**Table 4:** Preoperative and postoperative measurements for the orbit.

No (n = 14)	Preoperative Diff.	Postoperative Diff.	*P *value
Volume (mm^3^)*^,†^	3.93 (0.35–10.95)	1.05 (0.12–3.61)	0.001
Angle of TZ (degree)*^,†^			
Anterior 1/3	15.94 (0.1–89)	5.51 (0–30.1)	0.134
Middle	35.94 (0.5–144)	5.74 (0.3–21.4)	0.016
Posterior 1/3	41.18 (5.1–127.3)	5.15 (0–18)	0.002

*Diff* difference of the affected and the unaffected eye, *TZ* transition zone of orbital buttress; *Mean, ^†^Range.

### Case presentation

A 59-year-old female patient who met with a motorcycle accident presented with a right face contusion. Preoperative CT revealed a right zygoma tetrapod fracture with a concomitant right orbital floor blow-out fracture. Clinically, right malar depression with ecchymosis and subconjunctival hemorrhage were documented. No EOM limitation, diplopia, or visual acuity deficiency was noted. The patient had also sustained a Colle’s fracture of the right forearm, but she had no known systemic underlying disease.

**Figure 1:**
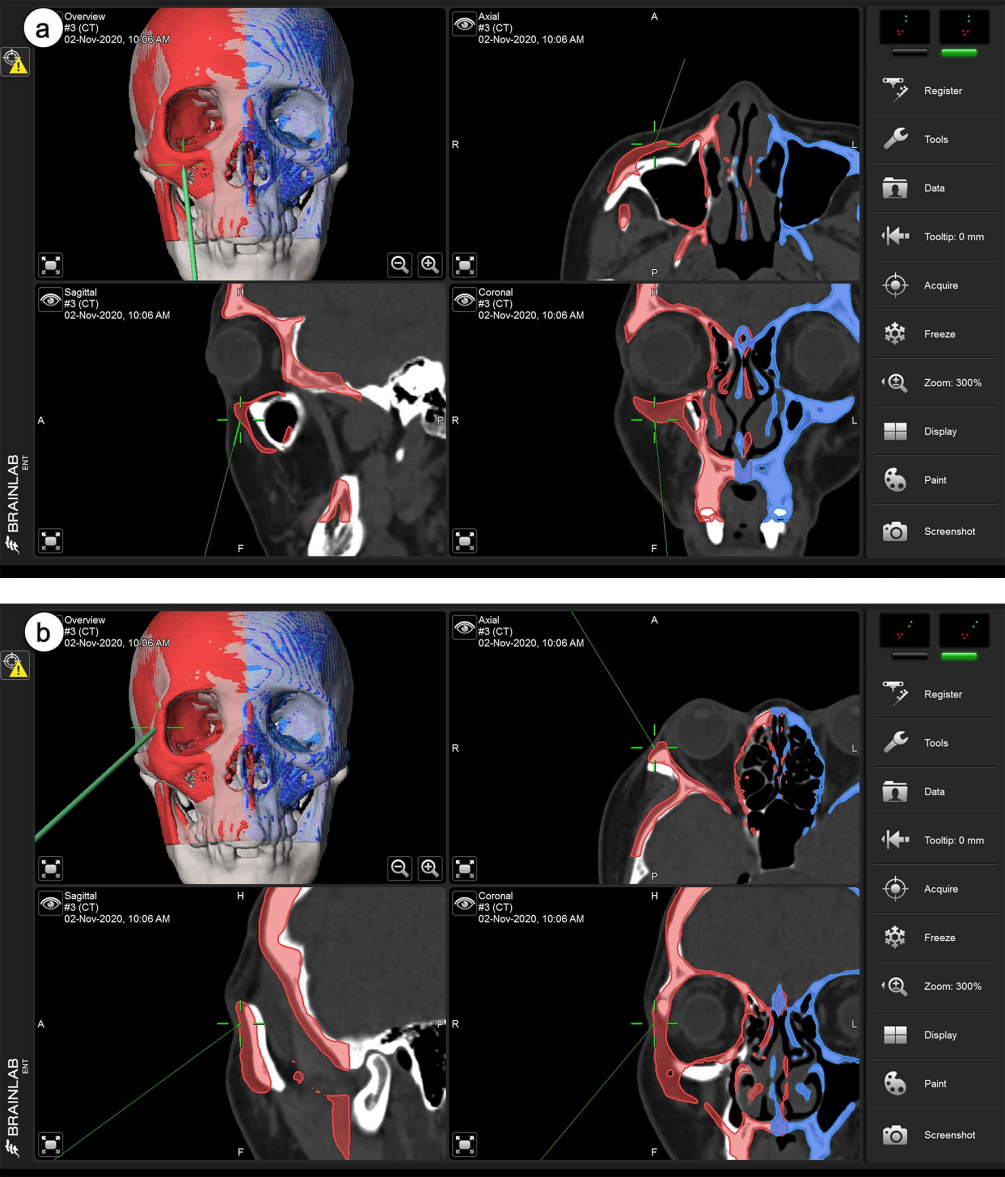

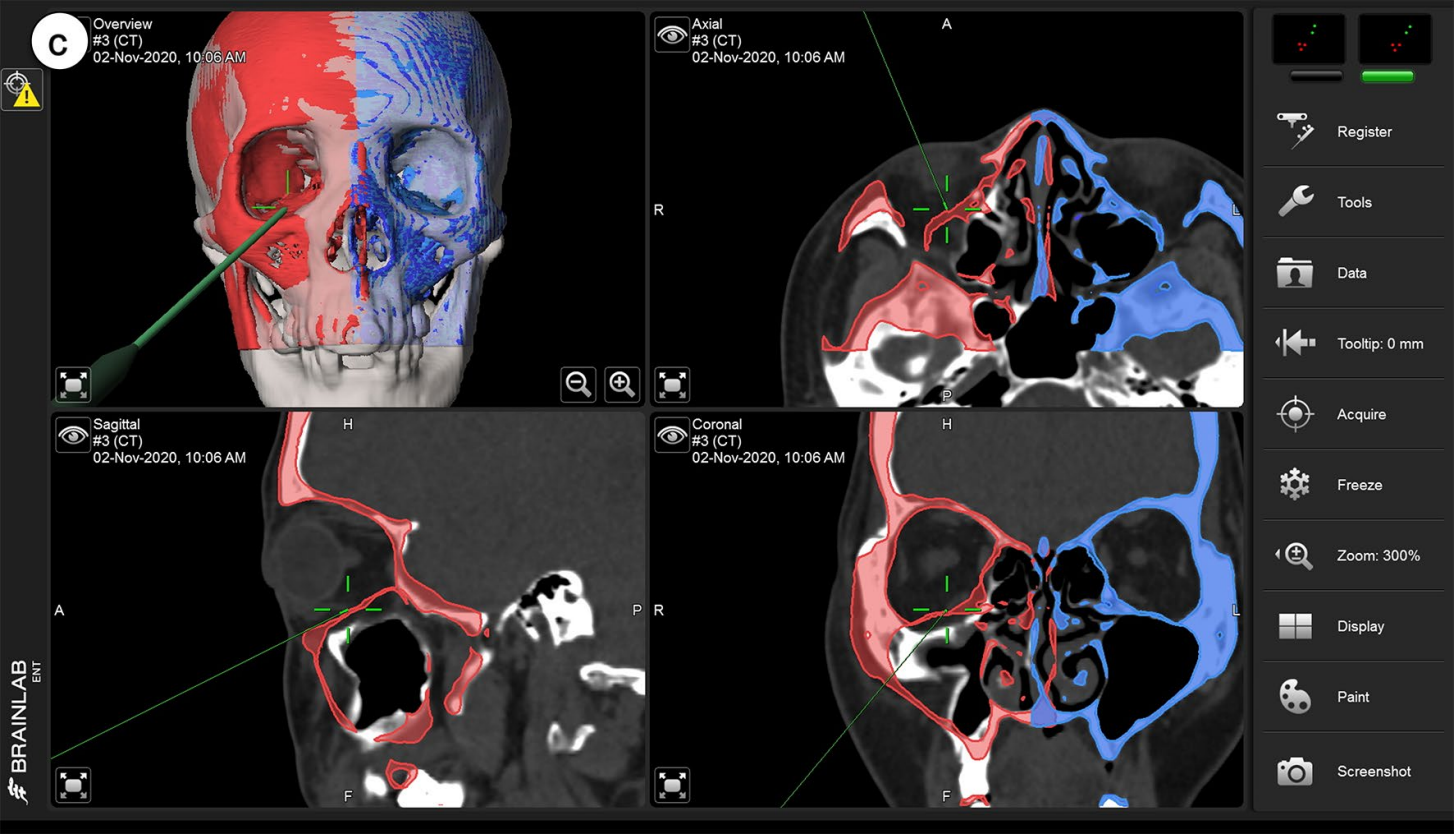
(**a**) Intraoperative real-time navigation for confirmation of zygomatic bone position at the zygomatic prominence. The semiautomated segmentation objects of the digital template were created on the unaffected side (blue) and superimposed onto the preoperative CT images, then they were mirrored to the affected side (red), resulting in an ideal presurgical plan with symmetrical positioning of the bones. The real-time position of the reduced zygoma prominence was confirmed by the laser pointer in the axial, coronal, and sagittal views. CT, computed tomography. (**b**) Intraoperative real-time navigation for confirmation of zygomatic bone position near the zygomaticofrontal junction. The real-time position of the reduced zygoma near the zygomaticofrontal junction was confirmed by the laser pointer in the axial, coronal, and sagittal views. (**c**) Intraoperative real-time navigation for confirmation of reconstructed implant position on the orbital floor. The real-time position of the reconstructed implant is shown, as verified on the orbital floor by the laser pointer in the axial, coronal, and sagittal views.

**Figure 2:**
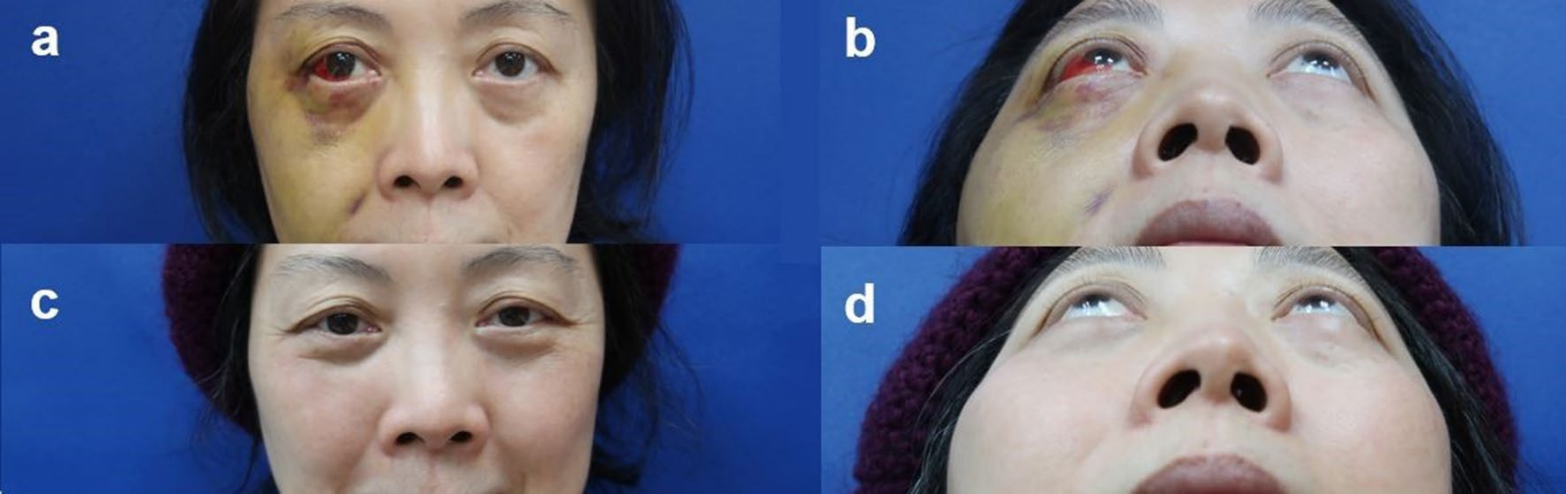
Clinical outcomes of facial symmetry and enophthalmos. The patient presented with right malar depression after zygomatico-orbital fractures in the preoperative frontal and axial views (**a**,**b**). Six months after surgery, the patient reported symmetric projection of both the eyeballs and bilateral malar eminence (**c**,**d**), and the results were esthetically satisfactory.

The ideal presurgical plan was designed by the BrainLAB software. Semiautomated segmentation volumetric objects were created on the unaffected side (blue) (Fig. 1), superimposed onto the preoperative CT images, and mirrored to the affected side (red). Under satisfactory general anesthesia, the head and neck area were prepared and draped in a sterile manner. Surface matching registration was conducted successfully by verification with laser pointer localization of the upper incisors. Then, right upper mucogingival and subciliary incisions were made, and the right zygoma-comminuted fracture and right orbital floor blow-out fracture were located. The bone was reduced under real-time surgical navigation guidance and fixed with mini plates over the ZF junction, infraorbital rim, lateral ZM buttress, and anterior maxillary wall. The orbital floor blow-out fracture was re-established with titanium mesh under navigation guidance. The real-time position of the reduced zygoma prominence and orbital floor was confirmed by the laser pointer in the axial, coronal, and sagittal views. Thereafter, intraoperative 3D C-arm CT was used to verify the final position, with imaging of the entire facial skeleton. The CT images showed satisfactory conformity with the presurgical design. The wound was repaired in layers. At the 6-month follow-up, the patient revealed symmetric bilateral malar and eyeball projections. No postoperative complications, such as diplopia, EOM limitation, enophthalmos, or hypoglobus, were reported (Fig. 2).

## Discussion

Our study results revealed that the use of the computer-assisted surgical navigation system for presurgical planning and intraoperative real-time guidance with 3D C-arm CT provides accurate and effective zygoma reduction and orbital fracture reconstruction. Among the eight patients with a facial fracture involving the zygoma, the postoperative sum of displacement in each zygomatic suture decreased from 19.48 to 1.96 mm and the DI decreased from 13.56 to 2.44 mm. The final distances between each zygomatic suture point or zygomatic prominence surface point and that of the presurgical plan was less than 2 mm, which is clinically undetectable appearance-wise.

All the patients had obvious preoperative facial asymmetry with malar depression or zygomatic arch protrusion. After the operation, facial symmetry improved in all patients. For orbit assessment, the orbital volume difference between the affected and unaffected eyes decreased from 3.93 to 1.05 mm^3^ after the operation. An increase in orbital volume causes enophthalmos. It has been reported that enophthalmos of < 2 mm is not clinically recognized^25–27^. The correlation between the orbital volume difference amount and enophthalmos degree ranges from 0.47 to 0.87 mm per 1-mm^3^ volume expansion^28–30^. Theoretically, the 1.05-mm^3^ volume difference is less likely to result in clinically significant enophthalmos.

Five of the fourteen patients with fractures involving the orbit presented with preoperative enophthalmos. Only one patient, who received secondary reconstruction, had residual mild enophthalmos. This may be related to the cicatricial effect and fat atrophy after inadequate primary manipulation of the orbital fracture^31^. The clinical outcomes may not be certainly predictable due to the underestimation of soft-tissue-related effect, even under adequate anatomical reconstruction^19^. For the angles of the transition zone of the orbital buttress, the middle and posterior one-third showed significant bilateral angle differences preoperatively and postoperatively. However, the difference in the anterior one-third only showed a decreasing trend. This may result from the small sample size and/or the fact that fractures of the medial wall and floor mostly affect the middle and posterior regions; thus, the bilateral angle difference of the anterior one-third was relatively less than those the other two locations, causing insignificant changes after the operation.

The role of patient specific implant (PSI) was well mentioned in literature. It allows accurate reconstruction in orbital fracture by the application of a digital workflow. Using the mirrored uninjured orbit as a template, the PSI was produced and placed under the guidance of intraoperative navigation system to ensure the ideal position in the orbit. The outcome is considered better than the manually bent titanium mesh implants^32^. Combination of surgical navigation, computer-aided surgical simulation (CASS) and PSI facilitates the planning and execution, improving the accuracy and precision of orbital reconstruction, especially in complex orbital fracture^33^. Recently, besides the usage of intraoperative imaging and navigation, the self-centering second-generation PSI was reported to provide even better accuracy and precision of implant positioning for quality control of reconstruction^34^.

Real-time surgical navigation systems have proved to be effective and beneficial in the clinical application of complex midfacial fracture reduction and complex orbital fracture reconstruction^35^. For reduction of the acute zygomatic fracture, under intraoperative navigation guidance, the deviation amount of each suture in the zygomatic fracture can be reduced to < 2 mm^11,36^. For orbital reconstruction, the rates of late and secondary surgery are more frequent than the rates for zygoma reconstruction, causing enophthalmos if left untreated. Correction is considered challenging if the fracture involves the transition zone of the orbital buttress. Previous studies have demonstrated the strong advantages of navigation guidance in improving precision and functional and aesthetic outcomes, facilitating intraoperative visualization, and avoiding vital structure injury in the re-establishment of extensive orbital fractures^10,37,38^. He et al. also reported that navigation-guided surgery with a 3D model and titanium mesh with Medpor implants are optimal for treating late ZMO fractures with severe enophthalmos^39^. In a recent study assessing extensive orbital reconstruction in a cadaveric model, real-time navigation was the fastest, most accurate, and safe method for orbital implant positioning^40^. Although navigation can be used to verify the position of implants or reduction in real-time, it cannot provide the most updated reconstructed bone information if the anatomy is changed during the surgery^41^. Therefore, the concomitant application of intraoperative 3D C-arm CT compensates for the inadequacy of the navigation system.

Intraoperative CT is also an effective tool for improving surgical outcomes of zygoma or orbital fractures. The important benefit of its usage is the integration of a wide flat-panel detector, which facilitates visualization of the entire anatomical craniofacial structure^14^. During the operation, it plays an important role in assessing the precision and accuracy of reduction, confirming the implant position in the orbit or the protrusion of the zygoma, and allowing immediate adjustment. In addition, the radiation exposure for midfacial imaging was much higher in the conventional multi-slice CT modality than in cone-beam CT systems^42^. Even for high-contrast structures, cone-beam CT could lessen the radiation exposure by about 50% of that used by conventional CT following particular parameters^14^. Using large-area flat-panel detectors, a detailed high-resolution 3D image of the craniofacial structure can be obtained^43^.

Using an intraoperative 3D C-arm system, Wilde et al. found that 4 of 21 patients with ZMO complex fractures had inadequate repair or reduction that required adjustment^44^. Chen et al. reported that 7 of 11 patients who underwent secondary or delayed primary orbital reconstruction with the aid of intraoperative CT required intraoperative adjustment^38^. Nonetheless, the avoidance of radiation exposure from postoperative imaging, surgical morbidity^44^, and another revision surgery exceeds the disadvantage of an increased operation time^17,23^.

Although intraoperative 3D C-arm CT ameliorates the surgical results in ZMO fractures^23^, it does not involve a real-time guidance because each implant position correction requires repeated intraoperative imaging, which take time to process. To sustain the advantages and eliminate the disadvantages of the surgical navigation system and intraoperative 3D C-arm CT, these two techniques were coupled for midface fracture reconstruction. In 2011, Scolozzi et al. reported the combination of “mirroring” computational presurgical planning, intraoperative surgical navigation system, and C-arm cone-beam CT with a flat-panel detector for management of primary and secondary midfacial fractures in five patients^45^. For further proof of the principle, our study quantitatively measured the surgical outcomes of parameters in the zygoma and the orbit, reflecting the standard usage of the navigation system as a real-time guide and the 3D C-arm CT as the final verification of the reconstruction results. With intraoperative CT alone, the correction rate during surgery can be as high as 63.3%^17^. When coupled with the navigation system and 3D C-arm CT, the intraoperative correction rate was 13.3%, which is much lower compared with that observed for CT alone during surgery.

However, surgical navigation system was more timing-consuming, and the technical requirements are demanding^46^. The time of steep learning curve should also be taken into account for device setup^47^ and presurgical planning, though it can be decreased by gaining more experience. The cost of navigation system and intraoperative CT were also not included in the health insurance, therefore the cost-effectiveness should be more concerned by patients. Nevertheless, the installation of navigation system brings additional injury on skull and intraoperative CT causes radiation. It would be more beneficial to use both of these techniques when facing the comminuted zygomatic fractures or extensive orbital floor and medial wall fractures that involved the transitional buttress. The surgical time can be offset by its accurate guidance for the optimal reconstructive position. For the simple fracture which the reconstruction landmark is still preserved, the use of both of these techniques may be superfluous.

In conclusion, the surgical navigation system combined with intraoperative 3D C-arm CT for the management of ZMO fractures has the advantages of both the techniques, thereby facilitating anatomical landmark reduction and functional reconstruction effectively, improving the accuracy and precision of surgery.

## Methods

### Patients

Fifteen patients (4 males and 11 females) were recruited between September 2020 and June 2021. The inclusion criteria were (1) unilateral ZMO fractures or unilateral isolated orbital (floor and medial wall)/zygoma fractures, either closed or open (2) The surgery and preoperative virtual planning were performed by a single surgeon (3) Patient who aged over 18 years old at the time of surgery (4) Able to write the informed consent. The exclusion criteria were (1) bilateral ZMO or orbital/zygoma fracture (2) The injury pattern combined with LeFort fractures (3) Orbital roof fracture (4) Patient who was pregnant (5) Patient who aged below 18 years old (6) Active malignancy of head and neck region. All the patients underwent ZMO reconstruction under the guidance of a surgical navigation system combined with intraoperative 3D C-arm (Ziehm Vision RFD 3D) (Ziehm Imaging GmbH, Nuremberg, Germany). When a patient’s first reconstruction was performed within 30 days of injury, it was defined as early or primary reconstruction, and reconstruction performed more than 30 days after the injury was defined as late or delayed primary reconstruction. Secondary surgery was defined when the patient had received previous reduction or reconstruction. Preoperative and postoperative signs and symptoms, including enophthalmos, hypoglobus, facial asymmetry, extraocular muscle movement (EOM) limitation, and diplopia were documented. This prospective study was approved by the Institutional Review Board of Chang Gung Memorial Hospital, Linkou Branch, Taiwan (IRB: 202000509A3C501). The procedures used in this study adhere to the tenets of the Declaration of Helsinki. Informed consent was obtained from all patients.

### Materials

**Figure 3:**
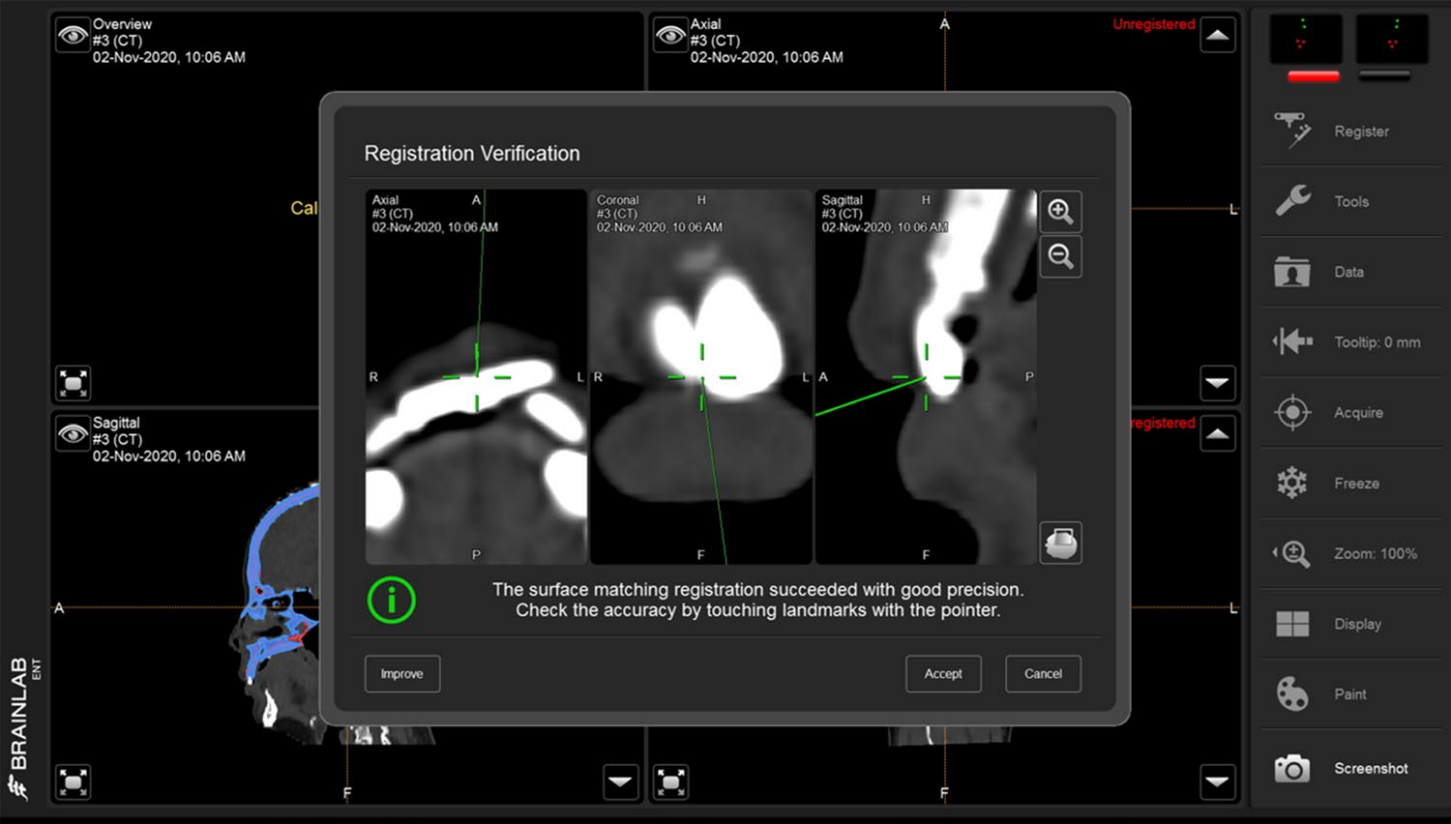
Presurgical verification of the accuracy of reduction. The laser pointer tip was placed on the surface and tip of the upper central incisors as landmarks to determine the precision of the anatomical position in three dimensions. A difference of < 1 mm between the preoperative imaging and the landmarks of the navigation pointer was considered acceptable.

We obtained preoperative computed tomography (CT) images of the facial bone on the day of surgery. The collimation/slice thickness was 1 mm. Digital Imaging and Communications in Medicine data was exported for further virtual planning and simulation in the 3D modeling software (BrainLAB AG, Munich, Germany). After loading the image data, the alignment of the cranium was adjusted according to the Frankfort Horizontal plane (FH) and vertical midline. The subsequential objects of the unaffected orbit and zygoma were created by semiautomated segmentation. The digital data was superimposed to the bone of the unaffected side and then mirrored to the affected side for surgical planning. We used the BrainLAB Vector Vision navigation platform as a guide to confirm the adequacy of zygomatic reduction or orbit reconstruction during operation. A tool adapter as skull reference was fixed to the parietal bone of the unaffected side. Three reflective plastic spheres with glass-grain coating (Latero Reference Star, BrainLAB) were secured to the adapter. A laser pointer (Z-touch BrainLAB) was used to surf and scan the frontal, periorbital, and nasal skin randomly for surface matching. When verifying the registration accuracy between CT images and the patient’s anatomical structure, we placed the navigation pointer tip on the surface of the upper central incisors as a landmark to check the precision of the anatomical position in three dimensions (Fig. 3). A difference of < 1 mm between the preoperative imaging and the landmarks of the navigation pointer was considered acceptable.

### Surgical procedure and intraoperative 3D C-arm

With the patient in the supine position under general anesthesia, the head and neck area were sterilized with a povidone-iodine solution, and the oral cavity was prepared with antiseptic gargles. According to the fracture patterns, incisions were made on the mucosa of the upper gingivobuccal sulcus for zygoma reduction or on the inferior conjunctiva or subciliary area for orbital reconstruction. To ensure precise reduction, the surgical navigation system was used to examine every suture line or critical point repeatedly until it matched the presurgical plan (Fig. 1). The MatrixMIDFACE Plating System was used for fixation of the fractured zygoma after reduction. A preformed mesh (DePuy Synthes Matrix MIDFACE Preformed Orbital Plates), titanium mesh, and Medpor (Porex Surgical Inc, Atlanta, Ga) were used for reconstruction of the fractured orbital floor and medial wall. In some patients, when the mesh alone was unable to ideally match the presurgical plan due to poor support by the surrounding bone, additional Medpor pieces were positioned under the mesh at the orbital buttress of the transition zone or at posterior ledge of the orbital floor.

**Figure 4:**
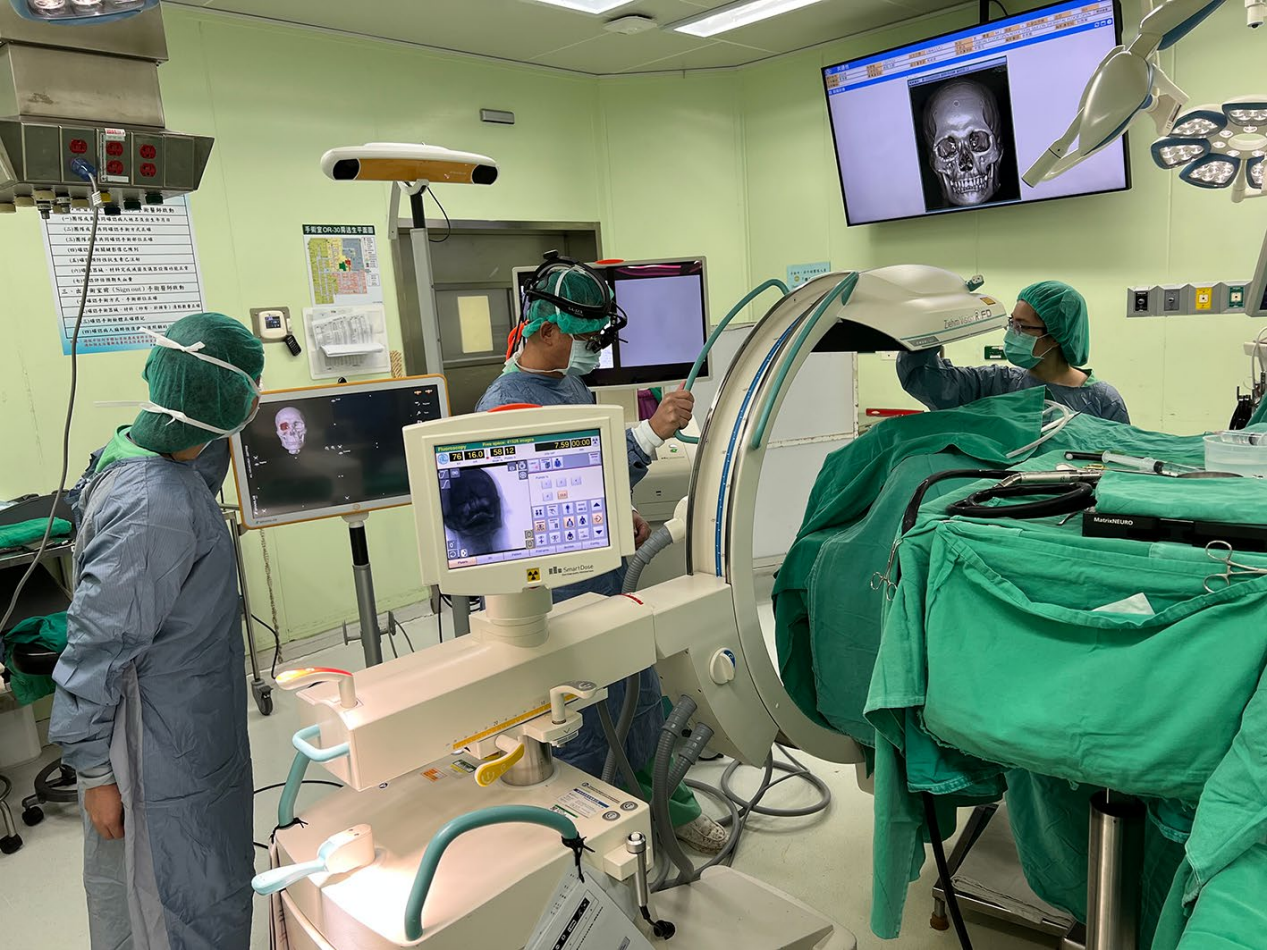
The setup of surgical navigation and intraoperative 3D C-arm computed tomography. The surgical navigation was used as a guide to determine the implant position. After plating, intraoperative 3D C-arm CT was performed to examine the adequacy of zygoma reduction and orbital reconstruction.

**Figure 5:**
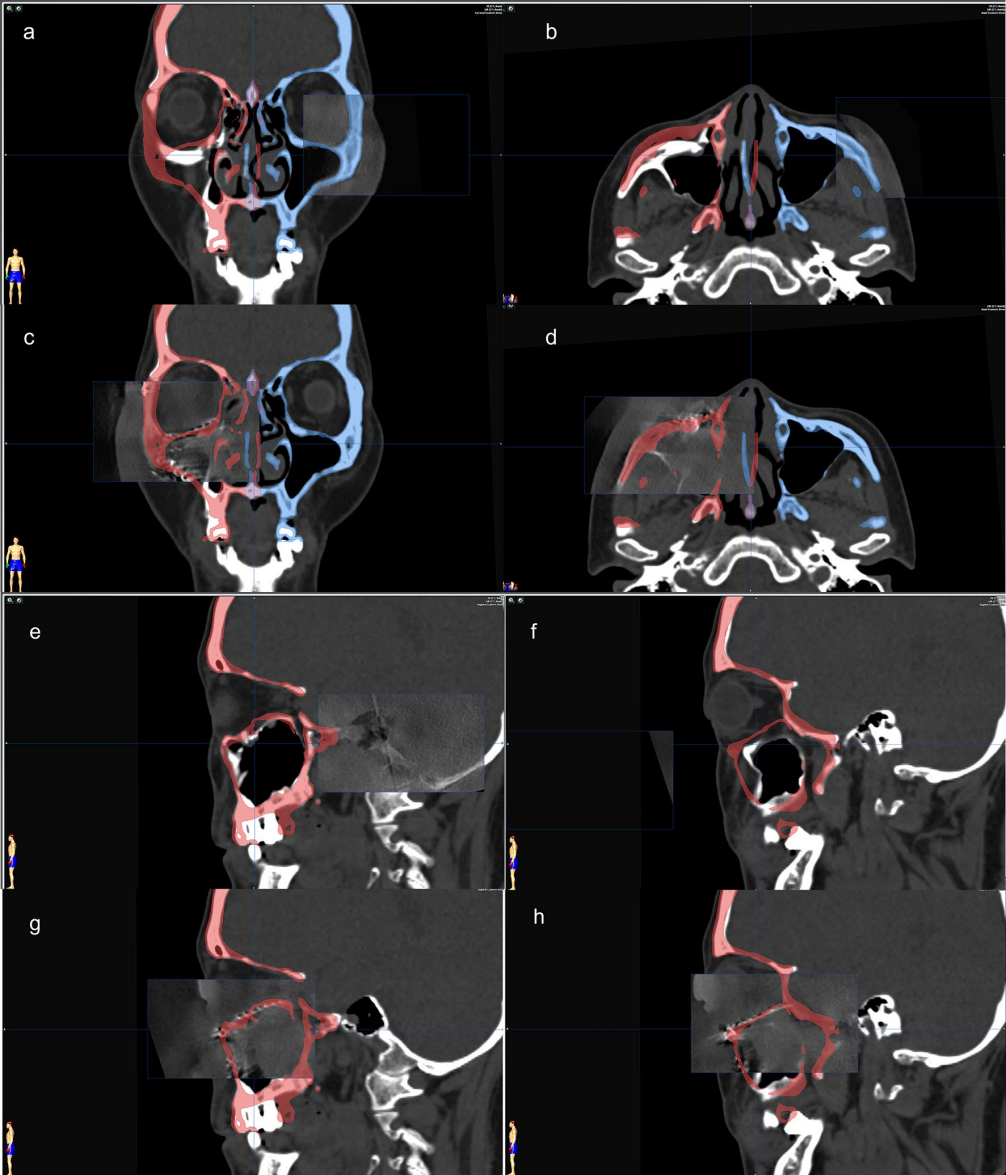
Fusion of preoperative computed tomography image, navigation presurgical planning, and intraoperative 3D C-arm computed tomography image. The intraoperative 3D C-arm computed tomography image is merged partially to the objects of navigation planning at the unaffected side (left) on the coronal and axial view (**a**,**b**). The updated position of the reduced zygoma and reconstructed orbital floor with implants (**c**,**d**). From different panels of the sagittal view, the preoperative images (**e**,**f**) and intraoperative images after implant positioning (**g**,**h**). The bone and implant contour accurately matched the presurgical planning.

**Figure 6:**
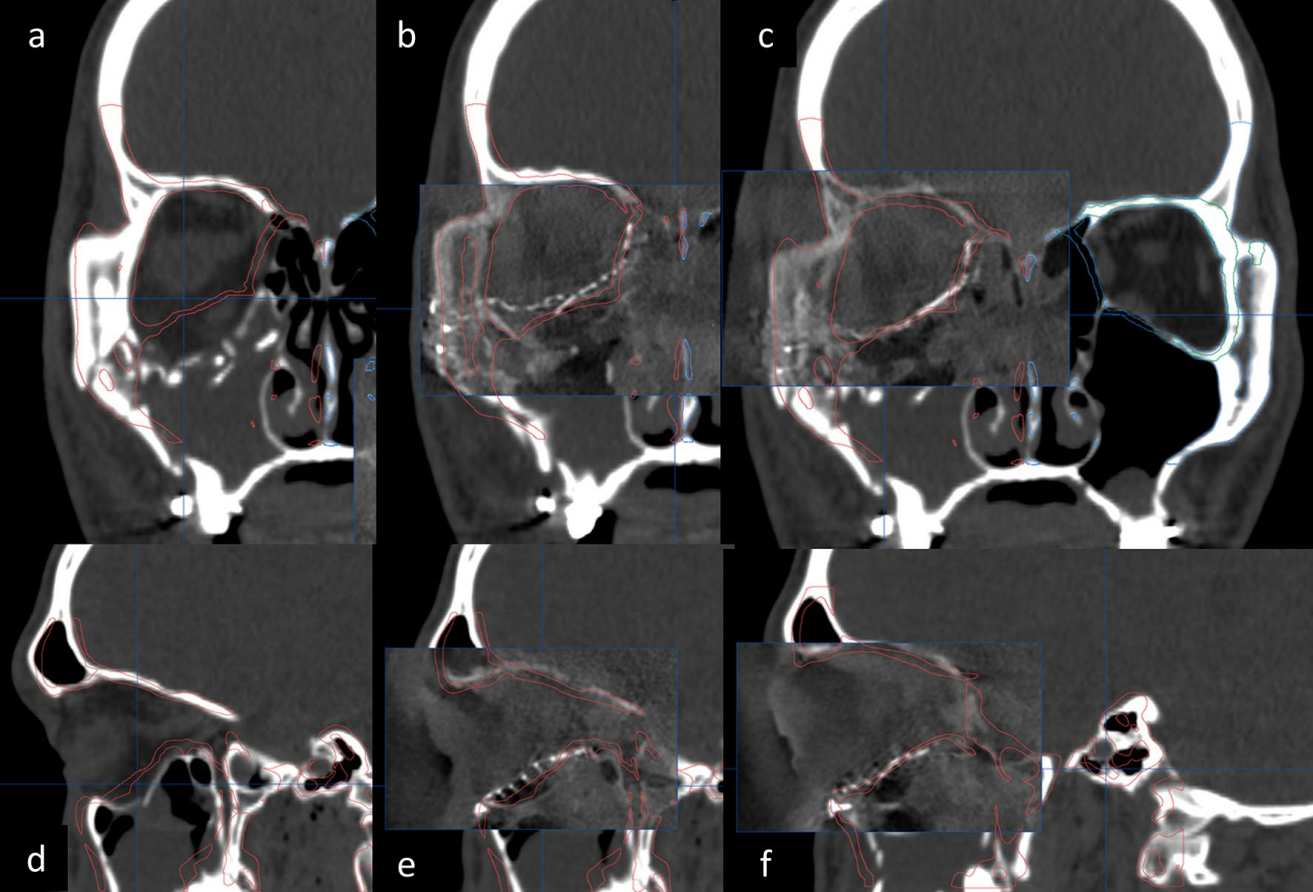
The implant adjustment of orbital reconstruction after intraoperative 3D C-arm computed tomography use. The right orbital floor fracture was shown on the coronal and sagittal view (**a**,**d**). After reconstruction, the 3D C-arm CT was applied, and the result revealed that the implant position was not in conformity with the presurgical plan (**b**,**e**). The adjustment was made for better reconstruction (**c**,**f**).

After plating, intraoperative 3D C-arm CT was performed to determine the adequacy of zygoma reduction and orbital reconstruction (Fig. 4). For assessment of the ZMO bone position, the flap panel detector 3D (low dose modifier) was used. An identical field of view was chosen to cover the entire skull bone. The uniform tube voltage of the 3D scan was set at 80 kV. The tube current [mA] was automatically dependent on the X-rayed object. The 3D scan images were acquired via a single rotation of the C-arm for 180° to obtain a complete dataset. The acquired images were immediately merged with the presurgical plan by the BrainLAB software to compare the conformity of the intraoperative reconstruction result and the plan (Fig. 5). Adjustments were then made as needed (Fig. 6).

### Zygoma measurement

**Figure 7:**
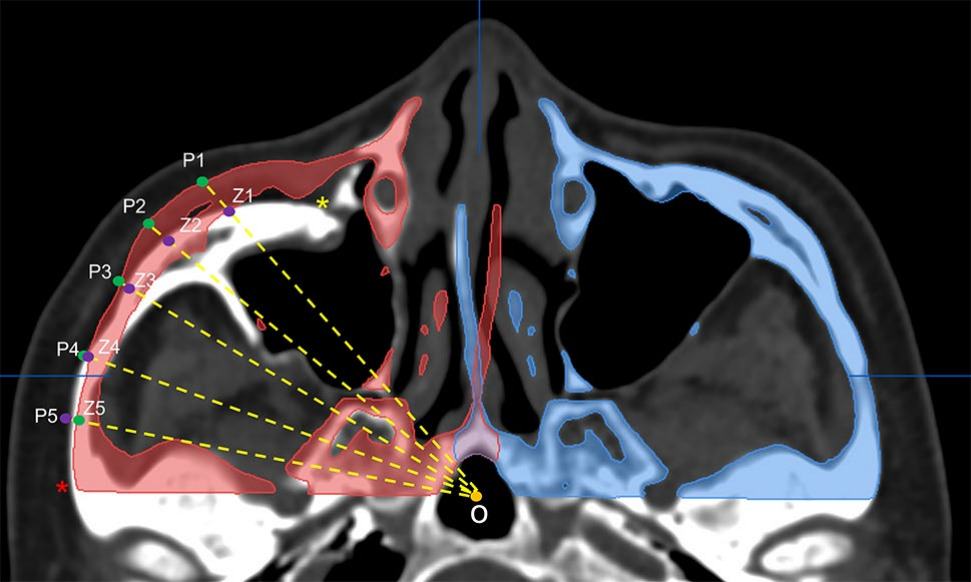
Measurements of zygoma conformity according to presurgical planning. The infraorbital foramen (yellow asterisk) and base of zygomatic arch (red asterisk) were identified on CT. The five points (Z1, Z2, Z3, Z4, and Z5) (violet points) on the zygomatic surface were defined and evenly distributed based on its angle of degree. The O point (yellow point) is defined as the intersection of the vertical midline and the horizontal line that passed through the bilateral zygoma arch base. The Z1O line (yellow dotted line) that intersected at the surface of the zygoma object during presurgical planning is defined as P1 (green point). The other points P2 to P5 (green points) are defined in the same manner. The preoperative and postoperative distances to virtual planning images between Z1 and P1, Z2 and P2, Z3 and P3, Z4 and P4, and Z5 and P5 were measured.

Using the facial bone CT image, the preoperative and intraoperative surgical landmarks were evaluated based on the degree of displacement at the following five key suture lines in the 2D fashion: the zygomaticofrontal (ZF), inferior orbital rim (IO), zygomaticosphenoidal (ZS), zygomaticomaxillary (ZM), and zygomaticotemporal (ZT)^48^. The bone displacement value of each suture line was summed for comparison of preoperative and postoperative findings. In the 3D fashion, a CT scan in the axial view parallel to the FH plane that displayed the entire zygoma shape was chosen for evaluation. The presurgical plan was displayed simultaneously. We identified the infraorbital foramen and the base of the zygomatic arch upon CT. Between these two landmarks, the five points (Z1, Z2, Z3, Z4, and Z5), which were evenly distributed based on the angle of degree, were defined on the surface of zygoma. We also defined the point (O) as the intersection of the vertical midline and the horizontal line that passed through the bilateral zygoma arch base. The presurgical planning, that is the object mirrored from unaffected side, was used as reference for measurements in the CT-dataset. The Z1O line that intersected at the surface of the zygoma object in presurgical planning was defined as P1. The other points, i.e., P2 to P5, were defined in the same manner. We measured the preoperative and postoperative distances between Z1 and P1, Z2 and P2, Z3 and P3, Z4 and P4, and Z5 and P5 on the same cut of axial view and defined the discrepancy of the summation of these five distances as the “deviation index” (DI) to indicate the difference between the actual zygoma position and the ideal surgical plan (Fig. 7). The distances of Z1O to Z5O and P1O to P5O were also measured and added up to determine the conformance between postoperative results and presurgical planning.

### Orbit measurements

**Figure 8:**
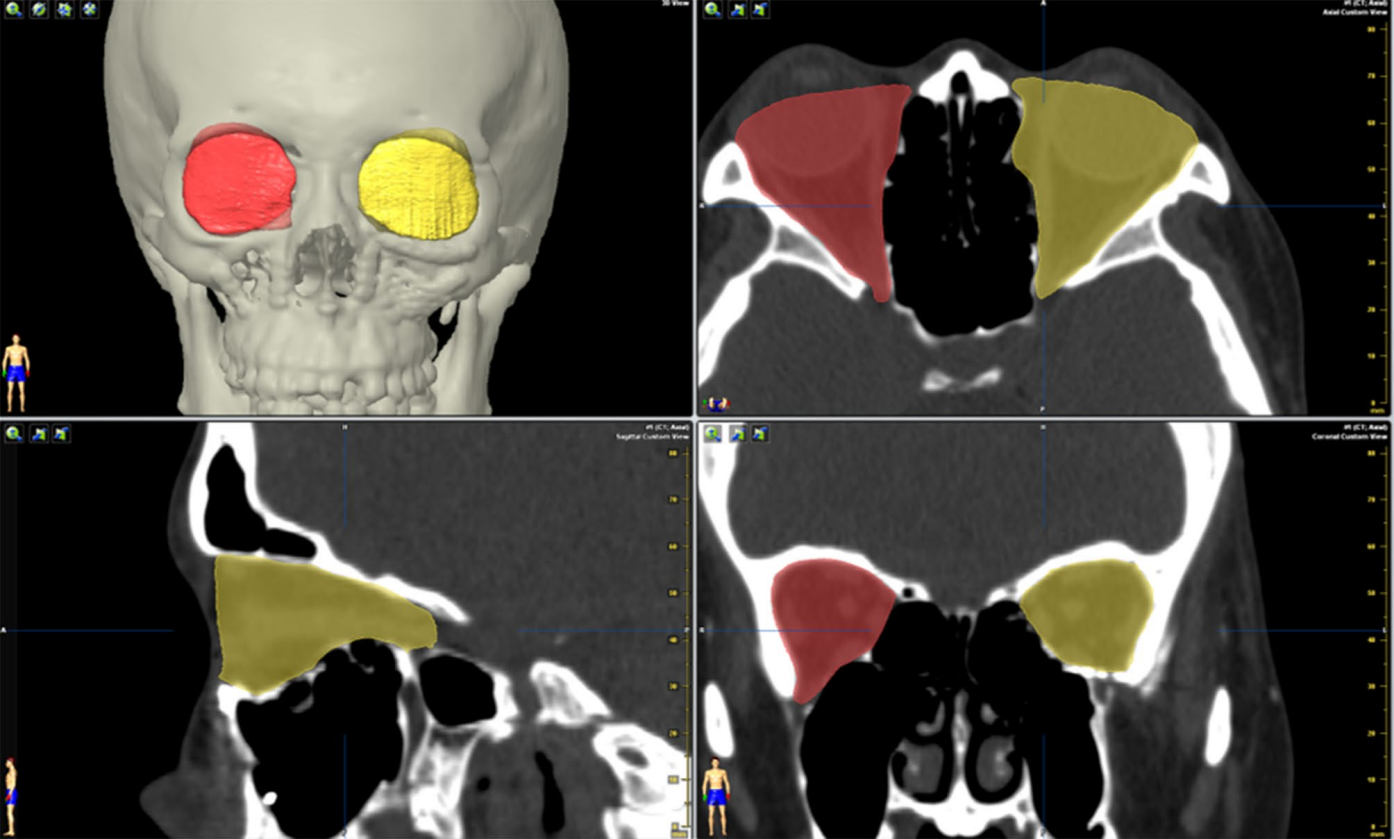
Measurements of bilateral orbital volume. The orbital volume was measured by the built-in function of object auto segmentation of the bilateral orbital cavity in the BrainLAB software. The fine adjustment of the object border was performed manually, and the orbital volume was obtained.

**Figure 9:**
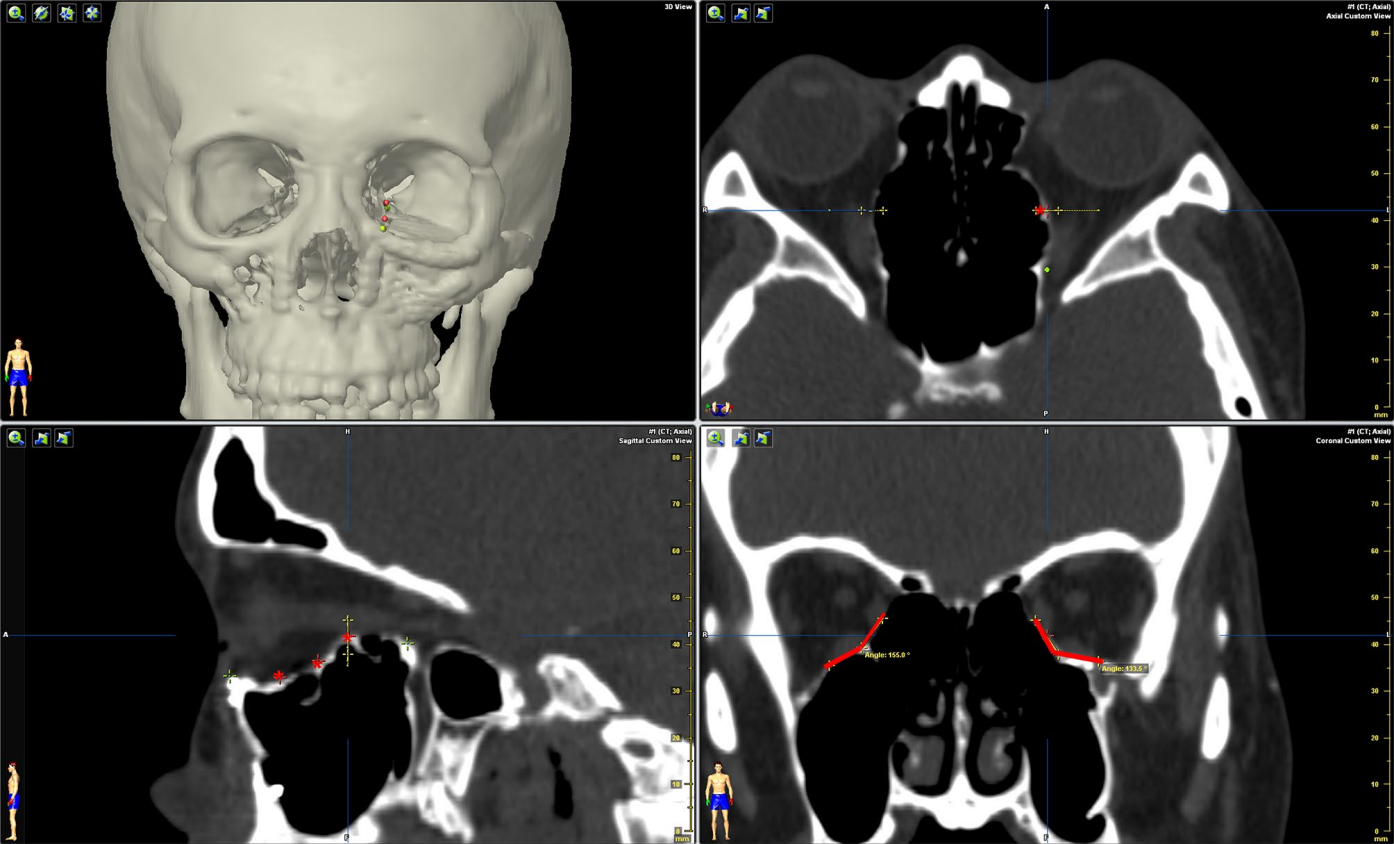
Measurements of bilateral angles of the transitional zone of the orbital buttress. The anterior, middle, and posterior points (red stars) are identified and evenly distributed on the orbital floor at the transitional buttress on the sagittal plane. The angles of the transitional buttress were measured on the coronal plane bilaterally. The figure shows that the bilateral angles of the posterior area are determined.

For the patient with fractures that involved the orbital floor and medial wall, the orbital volume and bilateral angles of the orbital buttress of transition zone at the anterior, middle, and posterior one-third were measured preoperatively and intraoperatively. The images were also assessed by the BrainLAB software. The head was aligned, as previously described. The objects of the bilateral orbital cavity were created by autosegmentation. Fine adjustment of the object's borders in the coronal, axial, and sagittal views was performed via an inbuilt function for optimizing the conformity to the bone margins. After shaping, the orbital volume of both the eyes was obtained (Fig. 8). For angle assessment of the transition zone of the orbital buttress, three points were identified on the orbital floor at the sagittal view. These three points equally divided the floor length and defined the plane of the anterior one-third, posterior one-third, and middle in the coronal view. The angles of the transition zone of the medial orbital buttress were measured from the coronal view and compared bilaterally (Fig. 9). The conformance of orbital parameters between the presurgical planning and postoperative result was also determined.

### Statistical analysis

Demographic data are described as number or mean (range, min–max). Preoperative vs postoperative data and presurgical planning vs postoperative results were compared using the Wilcoxon signed-rank test. The results represented statistical significance at a *P* value of < 0.05.

### Ethics approval

The study was approved by the Institutional Review Board of Chang Gung Memorial Hospital, Linkou Branch, Taiwan (IRB: 202000509A3C501). The procedures used in this study adhere to the tenets of the Declaration of Helsinki.

### Consent to participate

Informed consent was obtained from all individual participants included in the study.

### Consent for publication

Patients signed informed consent regarding publishing their data and photographs.

## Data Availability

All authors are sure that all data and materials support the published claims and comply with field standards.

## Code availability

All authors are sure that the software application supports the published claims and comply with field standards.
